# Chronic wasting disease: Emerging prions and their potential risk

**DOI:** 10.1371/journal.ppat.1006619

**Published:** 2017-11-02

**Authors:** Samia Hannaoui, Hermann M. Schatzl, Sabine Gilch

**Affiliations:** 1 Department of Ecosystem and Public Health, Faculty of Veterinary Medicine, University of Calgary, Calgary, Canada; 2 Calgary Prion Research Unit, University of Calgary, Calgary, Canada; 3 Department of Comparative Biology and Experimental Medicine, Faculty of Veterinary Medicine, University of Calgary, Calgary, Canada; Washington University School of Medicine, UNITED STATES

Prions cause fatal neurodegenerative diseases in humans and animals by converting the cellular prion protein PrP^C^ into aggregation-prone PrP^Sc^. Chronic wasting disease (CWD) is a prion disease or transmissible spongiform encephalopathy (TSE) of free-ranging and farmed cervids. CWD is highly contagious and transmitted through horizontal transmission enabled by the shedding of prions in excreta and their persistence in the environment. The disease is undergoing a dramatic spread across North America, has been found in South Korea, and, recently, has been identified for the first time in Europe in free-ranging reindeer (*Rangifer tarandus tarandus*) and moose in Norway.

## CWD

CWD first appeared in North America in captive animals in 1969 when biologists conducting a physiological study recognized that their animals often died with a syndrome of weight loss and behavioral changes. The biologists thought that the animals were probably dying of a nutritional deficiency due to toxin exposure or stressful conditions of captivity. In 1978, histological examination of brains of affected deer from wildlife research facilities in Colorado and Wyoming, United States, allowed us, for the first time, to recognize TSE features and define CWD as such [1]. Since then, CWD has had an impressive expansion and a very rapid spread in North America; the geographic range of CWD includes 21 US states and 2 provinces in Canada (Saskatchewan and Alberta) and is most likely to continue to grow [2]. Disease prevalence in free-ranging deer can be as high as 40% in the most endemic areas in Colorado and Wyoming [3]. More recently, CWD has been described in Northern Europe [4].

CWD is a slowly developing disease, with prolonged incubation periods. Experimental inoculation of mule deer has confirmed a 2- to 4-year incubation time [3]. The disease has been found in yearling deer and elk as well as in adult animals as old as 15 years. Clinical symptoms include physical and behavioral changes [5]. All these symptoms can be subtle early in the disease or fall within the normal repertoire of behavior or seasonal body mass fluctuations. Therefore, diagnosis based on clinical signs is not reliable and pathological or biochemical analyses of brain or lymphatic tissue are necessary. These will reveal neuronal vacuolation and spongiform changes, astrocytosis, and PrP^Sc^ accumulation in the brain or PrP^Sc^ deposits in lymphoid tissues.

In most prion diseases, PrP^Sc^ and infectivity are restricted mainly to the brain. However, in CWD, PrP^Sc^ can be found in many extraneural tissues, body fluids, and excreta, facilitating horizontal transmission.

## What species are susceptible to CWD infection?

The known natural hosts of CWD are elk (*Cervus canadensis*), mule deer (*Odocoileus hemionus*), white-tailed deer (*O*. *virginianus*), moose (*Alces alces*) [5], and reindeer [4]. Red deer (*C*. *elaphus*) [3] and fallow deer (*Dama dama*) [3] are also susceptible to CWD via experimental transmission. Interspecies transmission of CWD to noncervid animals has not been observed under natural conditions. However, due to the shared habitats of free-ranging cervids with other wildlife or domestic species in CWD endemic areas, there is increasing concern over the susceptibility of certain species, especially livestock, to CWD. In cattle, only intracerebrally but not orally inoculated animals developed prion disease [3]. Prion transmission is most effective via the less physiological intracerebral route; thereby, CWD can be transmitted experimentally to goats, sheep, rodents, mink, ferrets, and squirrel monkeys.

## From the human health perspective—Is CWD a matter of concern?

CWD is one of the most contagious prion diseases and the substantial presence in extraneural tissues; shedding of CWD prion infectivity in urine, feces, and saliva into the environment; and prion persistence for years are driving forces of CWD transmission [6]. Deer hunting and venison consumption are very common in North America. As the geographic distribution and case numbers of CWD are constantly growing [2], exposure of humans to CWD prions becomes more likely. To date, bovine spongiform encephalopathy is the only example of interspecies transmission of prion disease to humans [7]. The potential zoonotic transmission of CWD is an alarming issue and still an open question [3, 8].

Laboratory studies suggest that the risk of CWD transmission to humans is low. One group reported low conversion efficiency of human PrP^C^ by CWD PrP^Sc^ into the misfolded form using an in vitro amplification assay [9]. However, sole in vitro studies are not sufficient to assess the risk for humans exposed to CWD agents. Inoculation of “classical” CWD prions into transgenic mice overexpressing human PrP^C^ did not result in disease [3] but it is not known whether humans resist infection with all natural CWD strains. Transmission experiments employing nonhuman primates as infection models are a matter of debate. Squirrel monkeys were susceptible to CWD infection [3]. Inoculation via different routes of CWD prions into macaques, which have a prion protein (PrP) sequence that differs more from human PrP than that of squirrel monkeys although macaques are genetically closer to humans [10], is still a matter of debate [3]. We also can expect a long incubation period in nonhuman primates, as illustrated when sheep scrapie thought to be not zoonotic was transmitted to macaques [11]. With this in mind, studies in nonhuman primates are ongoing and it could take more than 10 years for the animals to develop disease. On the other hand, epidemiological studies did not show any correlation between CWD prion exposure and human prion disease, whether the cohort was large and population based [3] or small with case series [12–14]. During a routine surveillance over a period of 6 years (1993 to 1999) in Wyoming and Colorado, neither an overall increase in the incidence of Creutzfeldt-Jakob disease (CJD) [15] nor unusual prion disease subtypes or increased incidence in CJD patients who had regularly consumed venison [16, 17] was observed.

These findings suggest a notable species barrier between cervids and humans; however, prion diseases are dynamic; interspecies passage of CWD can result in prion adaptation to new host species. Besides, the existence of more than one CWD strain [18] may contribute to higher heterogeneity in disease and transmission profiles [19].

## Can the species barrier for CWD to humans be crossed?

Although the evidence gathered so far is in favor of a low risk to transmit CWD to humans, results from in vitro studies indicated that the species barrier is not absolute.

We know now that the species barrier is not only regulated by the PrP primary structures of donor (PrP^Sc^) and recipient (PrP^C^) and the importance of compatibility between the 3-dimensional shapes of PrP^Sc^ and PrP^C^ becomes more evident. Prion strains differ in PrP^Sc^ conformation, and new CWD strains may emerge through prion adaptation to new species and/or passage through cervids expressing different PrP genotypes.

Prion adaptation is characterized by shortened incubation time, increase of attack rate, and changes in PrP^Sc^ properties and deposition profile upon serial passages in a new species after cross-species transmission [20]. New prion strains therefore might gradually develop if CWD prions are able to transmit and propagate in a new species. This may be CWD-susceptible rodents such as vole species that live in CWD endemic areas. Upon environmental retransmission of such a putative “intermediate host-derived CWD,” the species barrier between human PrP and the new PrP^Sc^ conformer may be obliterated. Because of the long time required between exposure to CWD agents and the development of prion disease, many years of continuous surveillance are necessary to be able to say what the risk, if any, of CWD is to humans.

## Can we stop CWD?

Even though management policies for captive animals through quarantine and depopulation of CWD-affected herds appear efficient, attempts to control CWD even in confined facilities failed because of persistence of infectivity in the environment.

Managing CWD in free-ranging animals is an even bigger challenge. CWD will continue to expand in North America and other territories, exemplified by its unexpected emergence in Norway. The absence of an effective vaccine despite considerable efforts to develop this strategy makes complete eradication of CWD not realistic to date. Besides, long incubation periods, subtle early clinical signs, a resilient infectious agent in the environment, and incomplete understanding of transmission all constrain options for controlling CWD. Norwegian authorities decided to cull an entire herd of 2,500 reindeer and to prevent migration into this area where CWD was found as an attempt to stamp out the disease [21]. Less drastic but similar approaches were taken in North America with targeted culling of deer to reduce population densities and thereby minimize the risk of transmission. However, success was limited and, ultimately, environmental reservoirs of CWD prions may sustain disease incidence [3].

Inherent difficulties in managing infected herds and premises underscore the need for aggressive surveillance to prevent introduction of infected carcasses to the human food chain.

Developing new noninvasive and preclinical diagnostic tools for live animals, e.g., by sampling their feces [22], could help to delimit infected areas. Selecting resistant genotypes for breeding farmed cervids could reduce CWD as has been demonstrated for scrapie in sheep.

In summary, extensive research to learn more about CWD strain variability and transmission properties is necessary to predict the risk for humans. Effective vaccine strategies and decontamination of infected facilities and environmental reservoirs need to be developed to counteract the spread of CWD.
